# New Anti-Cancer Strategy to Suppress Colorectal Cancer Growth Through Inhibition of ATG4B and Lysosome Function

**DOI:** 10.3390/cancers12061523

**Published:** 2020-06-10

**Authors:** Yuanyuan Fu, Qianqian Gu, Li Luo, Jiecheng Xu, Yuping Luo, Fan Xia, Fanghai Han, Liang Hong, Xiao-Ming Yin, Zhiying Huang, Min Li

**Affiliations:** 1 School of Pharmaceutical Sciences, Sun Yat-sen University, Guangzhou 510006, China; fuyy6@mail2.sysu.edu.cn (Y.F.); guqq@mail2.sysu.edu.cn (Q.G.); luoli26@mail2.sysu.edu.cn (L.L.); xujch6@mail2.sysu.edu.cn (J.X.); luoyp26@mail2.sysu.edu.cn (Y.L.); xiaf6@mail2.sysu.edu.cn (F.X.); hongliang@mail.sysu.edu.cn (L.H.); 2 Department of Gastrointestinal Surgery, Sun Yat-sen University, Guangzhou 510120, China; fh_han@163.com; 3 Department of Pathology and Laboratory Medicine, Tulane University School of Medicine, New Orleans, LA 70112, USA; xmyin@tulane.edu

**Keywords:** ATG4B, autophagy, colorectal cancer, dual-function inhibitor, lysosome inhibition, new anti-cancer strategy

## Abstract

Autophagy inhibition has been proposed to be a potential therapeutic strategy for cancer, however, few autophagy inhibitors have been developed. Recent studies have indicated that lysosome and autophagy related 4B cysteine peptidase (ATG4B) are two promising targets in autophagy for cancer therapy. Although some inhibitors of either lysosome or ATG4B were reported, there are limitations in the use of these single target compounds. Considering multi-functional drugs have advantages, such as high efficacy and low toxicity, we first screened and validated a batch of compounds designed and synthesized in our laboratory by combining the screening method of ATG4B inhibitors and the identification method of lysosome inhibitors. ATG4B activity was effectively inhibited in vitro. Moreover, 163N inhibited autophagic flux and caused the accumulation of autolysosomes. Further studies demonstrated that 163N could not affect the autophagosome-lysosome fusion but could cause lysosome dysfunction. In addition, 163N diminished tumor cell viability and impaired the development of colorectal cancer in vivo. The current study findings indicate that the dual effect inhibitor 163N offers an attractive new anti-cancer drug and compounds having a combination of lysosome inhibition and ATG4B inhibition are a promising therapeutic strategy for colorectal cancer therapy.

## 1. Introduction

Autophagy is a lysosomal recycling mechanism that is responsible for the degradation of cellular protein aggregates and damaged organelles [1]. Autophagic degradation occurs in the autolysosome and it is aided by lysosomal hydrolytic enzymes [2]. The recycling capacity of autophagy is involved in both physiological and pathophysiological conditions, and dysregulated autophagy has been linked to various human diseases. Of particular importance is the role of autophagy in cancer, since autophagy has been linked to playing a context-dependent role in cancer. Previous studies have indicated that autophagy prevents tumor growth at the initiation stages. Conversely, autophagy enables the survival of established tumors at the later stages from stressful conditions by increasing the autophagy flux [3,4,5]. Therefore, autophagy inhibition could be an effective way to control cancer development. 

Autophagy is a multi-step process and the majority of these steps are potential targets for autophagy regulation. There is evidence that autophagy inhibition can be effective in synergizing with chemotherapy to suppress tumor cell growth [6,7,8]. Currently, chloroquine (CQ) and the related hydroxychloroquine (HCQ) are the most widely used autophagy inhibitors in preclinical studies or clinical trials for cancer treatment [9,10,11]. Potent lysosome inhibitors such as Lys05 and VATG-032 have also been reported to inhibit autophagy and impair cancer cell growth in preclinical studies [12,13]. However, such lysosome inhibitors may not inhibit autophagy, but this could be attributed to the deacidification of lysosome or impaired lysosomal enzymatic function, which could cause unfavorable side effects [9,14]. Therefore, the development of more specific autophagy inhibitors is important for cancer treatment. 

ATG4B is the main cysteine protease which cleaves newly synthesized pro-LC3 and breaks down LC3 from PE, an essential protein for autophagosome elongation and maturation [15]. The association of ATG4B with cancer has recently gained much attention, due to its key role in the regulation of autophagy and cell differentiation. ATG4B has been reported as an oncogene in various cancer cells, including osteosarcoma, chronic myeloid leukemia (CML), human epidermal growth factor receptor 2 (HER2)-positive breast, and colorectal cancer cells [16,17,18,19]. Therefore, ATG4B may be a potential drug target for cancer and inhibition of ATG4B activity may serve as a new mean for cancer therapy. However, only a limited number of ATG4B inhibitors with a weak inhibition ability are available, which limit its clinical application.

Multitarget-directed ligands (MTDLs) is a concept where small organic molecules are designed for multifunctional effects [20]. In the last decade, the majority of multifunctional strategies have focused on treating complex diseases such as neurodegenerative diseases [21,22] and cancer [23]. Considering the single target inhibitors of either lysosome or ATG4B have poor effect, it will be a better therapeutic strategy to find the inhibitor of dual-function to lysosome and ATG4B. In this study, by establishing the screening method of ATG4B inhibitors, combined with the identification method of lysosome inhibitors, we first screened and validated a batch of compounds designed and synthesized in our laboratory, and we found a novel compound named 163N. In vitro, 163N could efficiently inhibit ATG4B activity. There was also suppression of autophagy by 163N, which mainly affected the function of the lysosomes, and also reduced the degradation of autolysosomes without affecting the fusion of autophagosomes with lysosomes. Furthermore, 163N was effective in attenuating the growth of colorectal cancer HCT116 cells in vitro and in vivo, and in suppressing autophagy in vivo. These study findings demonstrate that the novel dual-function autophagy inhibitor 163N showed excellent antitumor efficacy both in vitro and in vivo and might be a promising lead compound for future autophagy-based multi-functional drug discovery. 

## 2. Results

### 2.1. Discovery of a Potential Dual Functional Tumor Inhibitor

To weaken the acidity of lysosome inhibitors, to reduce the adverse effect of anti-lysosomal agents, and to improve the antitumor effect of ATG4B inhibitors, we first performed a fluorescence resonance energy transfer (FRET)-based screening for ATG4B inhibitors, using an in-house new drug library of 560 compounds, from which 15 compounds were screened out with IC50 below 50 µM (Table 1). Among these compounds, XOS00163, XOS00201, XOS00207, and 163N with a 2-phenylbenzofuran structure exhibited a strong inhibition effect on ATG4B with IC50 of 6.2 µM, 8.98 µM, 2.31 µM, and 17.82 µM, respectively (Table 1). Then adherent cells HeLa and HCT116, and suspension cells HL60 were analyzed by cell counting kit-8 (CCK-8) assay, and the results showed that only XOS00163, XOS00201, XOS00207, and 163N could reduce the cell viability of HeLa, HCT116 and HL60 cells, and 163N exhibited the best anticancer effect (Table 1). However, there was no growth arrest when the cells were cultured with other screened ATG4B inhibitors (Table 1). These findings demonstrated that the structure of 2-phenylbenzofuran is a good pharmacophore for developing therapeutic agents against ATG4B in cancer therapy. 

**Figure 1:**
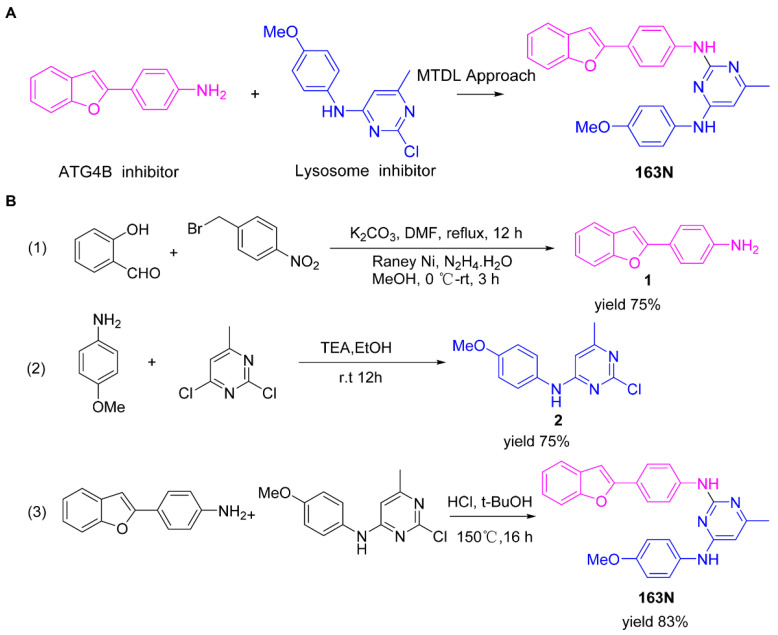
Graphical synthetic route of 163N. (**A**) The structure of 163N carries both 2-phenylbenzofuran and 6-methylpyrimidine-2,4-diamine. (**B**) Synthesis of 163N.

Moreover, we found that 163N bearing the structure of both 2-phenylbenzofuran and 6-methylpyrimidine-2,4-diamine. Our previous results implied that compounds with a 6-methylpyrimidine-2,4-diamine structure could cause lysosome inhibition [24]. We synthesized a series of compounds with a 6-methylpyrimidine-2,4-diamine structure (Figure S1A). As shown in Figure S1B, treatment with this series of compounds resulted in significantly quenching of red fluorescence, even stronger than bafilomycin A1 (Baf)-treated cells, suggesting that this series of compounds had the ability to efficiently increase the pH of lysosome. Next, we further monitored the degradation function of lysosome by dequenched bodipy-conjugated BSA (DQ-BSA assay). As shown in Figure S1C, similar to Baf or E64D plus pepstatin A treatment group, the red fluorescence signal in cells treated with this series of compounds was very weak, demonstrating that the proteolytic activity of lysosomes was affected by this series of compounds. Overall, the data indicate that this series of compounds are lysosome inhibitors. Thereafter, 163N bearing the structure of both 2phenylbenzofuran for ATG4B and 6-methylpyrimidine-2,4-diamine for lysosome might be a potential dual functional tumor inhibitor. The structure and the synthetic route for 163N are shown in Figure 1. 

### 2.2. 163N Inhibits the Activity of ATG4B

**Figure 2:**
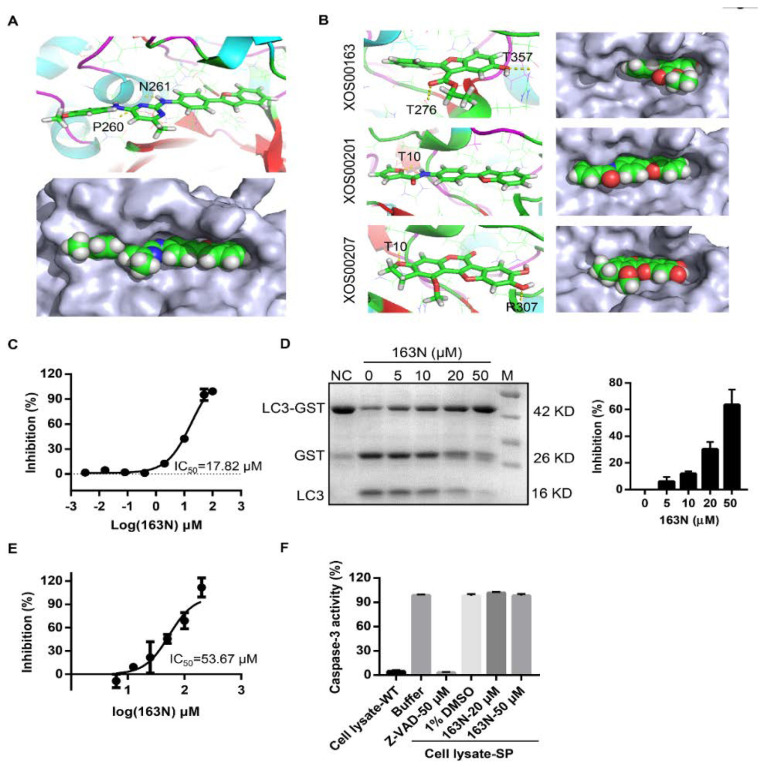
163N inhibits the activity of ATG4B. (**A**) Predicted binding model of compound 163N binds to ATG4B (Protein Data Bank (PDB) ID: 2CY7) pocket. 163N can form hydrogen bonds with residues Pro260 and Asn261 of ATG4B (yellow dotted lines). (**B**) Predicted binding model of compound XOS00163, XOS00201, and XOS00207 bind to ATG4B (PDB ID: 2CY7) pocket. (**C**) IC50 calculation of 163N for ATG4B from fitted curve by fluorescence resonance energy transfer (FRET) assay. (**D**) ATG4B (1 µg/mL) was incubated with different concentrations of 163N (5, 10, 20, 50 µM) at 37° for 30 min, then LC3B-GST was added to the mixture and incubated at 37° for another 30 min. SDS-PAGE was used to detect the inhibitory effect of 163N. The inhibition rate (%) was calculated based on the band density and quantified. Data are presented as mean ± SEM from three individual experiments. (**E**) Measurement of the IC50 of 163N for ATG4A by FRET assay. (**F**) Measurement of the specificity of 163N against other cysteine proteases. HeLa cells were treated with 1 µM of staurosporine (SP) for 12 h to induce cell apoptosis. Then, 2 µg of the whole cell lysate were mixed with indicated compounds at 37° for 30 min, Ac-DEVE-AFC (25 µM) were then added to a volume of 50 µL, and the emission of 505 nm for AFC was recorded.

Docking experiments using the closed-form of ATG4B (Protein Data Bank (PDB) code 2CY7) were first done to confirm whether 163N had any effects on the activity of ATG4B, as previously reported [25]. As shown in Figure 2A, we found that 163N could fit into the docking pocket (Figure 2A, lower panel), and was able to form two hydrogen bonds with Pro260 and Asn261. These are the two key residues of the regulatory loop (residues 259-262) covering the entrance of the catalytic site and undergoing a large conformational change when ATG4B interacts with LC3 [26]. FRET-based assay showed that 163N could dose-dependently suppress the activity of ATG4B with an IC50 of 17.82 µM (Figure 2C), which was slightly higher than that of XOS00163, XOS00201, and XOS00207 (Table 1). The rankings for these four compounds were predicted using docking results, and they were consistent with the IC50 data, possibly due to the limited pocket size of free ATG4B as 163N has a larger structure than the other three compounds. The docking results of XOS00163, XOS00201, and XOS00207 are shown in Figure 2B. A gel-based assay using substrate LC3-GST was also performed and 163N displayed a dose dependent inhibition effect on ATG4B activity (Figure 2D). We then tested whether 163N could also inhibit ATG4A activity. We found that 163N exhibited a weak inhibition effect on ATG4A with an IC50 of 53.67 µM by FRET-based assay (Figure 2E). In addition, we also tested the selectivity of 163N on caspase-3, another cysteine protease. As shown in Figure 2F, in contrast to the pan-caspase inhibitor Z-VAD-fmk, 163N had no inhibition effect on caspase-3 activity, even at a concentration of 50 µM. Together, these results suggest that 163N could inhibit the activity of ATG4B.

### 2.3. 163N Suppresses Autophagy Flux

**Figure 3:**
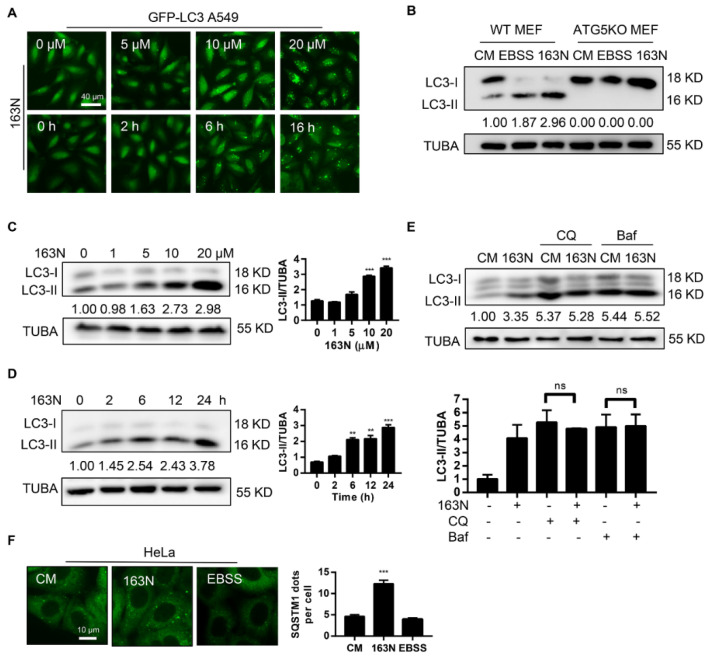
163N inhibits autophagic flux. (**A**) A549 cells expressing GFP-LC3B were treated with 163N for 6 h at different concentrations or treated with 163N (10 µM) over a certain time course, then the distribution of GFP-LC3B was examined. (**B**) Wild type (WT) Mouse Embryonic Fibroblast cells (MEFs) or ATG5KO MEFs were treated with 163N (10 µM) for 6 h, or EBSS for 2 h for western blotting assay. (**C**) HCT116 cells treated with different concentrations of 163N (1, 5, 10, 20 µM) for 6 h were analyzed by western blotting for endogenous LC3B. The ratio of LC3B-II/TUBA was calculated using ImageJ software. (**D**) HCT116 cells treated with 163N (10 µM) over a certain time course were analyzed by western blotting for endogenous LC3B. The ratio of LC3B-II/TUBA was calculated using ImageJ software. (**E**) HeLa cells were treated with 163N (10 µM) with or without CQ (40 µM) or bafilomycin A1 (Baf) (0.5 µM) for 6 h, and then endogenous LC3B was analyzed by western blotting. The ratio of LC3B-II/TUBA was calculated using ImageJ software. (**F**) HeLa cells were treated with 163N (10 µM) for 6 h, or EBSS for 2 h, and then immunostaining was used to detect SQSTM1 dots, and the number of SQSTM1 dots was quantified. The LC3-II intensities in relation to TUBA are shown in this Figure, and the uncropped blots and molecular weight markers are shown in Figure S7. Data are presented as mean ± SEM from three individual experiments. ** *p* < 0.01, *** *p* <0.001, ns, not significant.

To identify the impact of 163N on autophagy, A549 cells stably expressing GFP-LC3 were used. We found that 163N caused the accumulation of GFP-LC3 dots in dose- and time-dependent manners (Figure 3A). We then examined the effect of 163N on LC3 conversion in both HeLa and HCT116 cells, western blot analysis showed that 163N treatment resulted in dose- and time-dependent accumulation of LC3-II in both cell lines (Figure 3C,D and Figure S2A,B). However, similar to Earle’s Balanced Salt Solution (EBSS) treated cells, 163N treatment did not induce LC3-II in ATG5-deficient cells (Figure 3B), an indication that 163N-induced LC3-II was autophagy-dependent. To confirm the effect of 163N on autophagy, two autophagy inhibitors CQ and Baf were used to measure LC3-II turnover. Western blot analysis showed that 163N treatment did not further enhance the expression level of LC3-II induced by CQ or Baf groups (Figure 3E), therefore, 163N treatment may inhibit autophagy flux. An autophagy flux analysis in GFP-LC3 A549 cells was consistent with the western blot analysis (Figure S2C). In addition, in contrast to the EBSS treated cells, 163N significantly attenuated the degradation of the autophagic substrate SQSTM1 (Figure 3F), suggesting that 163N is a potent autophagic flux inhibitor.

### 2.4. 163N Increases the Accumulation of Autolysosomes

**Figure 4:**
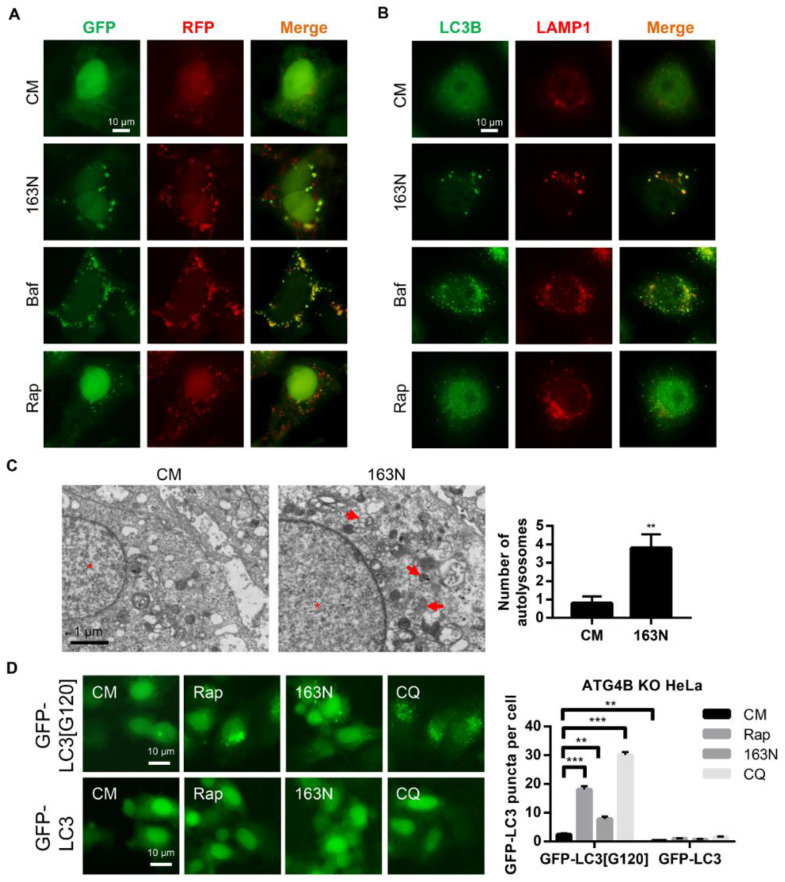
163N increases the accumulation of autolysosomes. (**A**) HEK293A cells expressing green fluorescent protein (GFP)-red fluorescent protein (RFP)-LC3 were treated with 163N (10 µM), rapamycin (Rap) (1 µM) or Baf (0.5 µM) for 6 h, then the colocalization of GFP and RFP puncta was detected. (**B**) HeLa cells were treated with 163N (10 µM), Rap (1 µM) or Baf (0.5 µM) for 6 h, then immunostaining was used to detect LC3B and LAMP1. The colocalization of LC3B and LAMP1 was measured. (**C**) HeLa cells were treated with 163N (10 µM) for 6 h, then transmission electron microscopy (TEM) was used to detect the ultrastructure of HeLa cells. Red arrows indicate normal autolysosome structures. (**D**) ATG4BKO HeLa cells expressing GFP-LC3[G120] or full length GFP-LC3 were treated with Rap (1 µM), 163N (10 µM), or CQ (40 µM) for 6 h, then the distribution of GFP-LC3 was examined. And the number of GFP-LC3 dots were quantified. Data are presented as mean ± SEM from three individual experiments. * *p* < 0.05, ** *p* < 0.01, *** *p* < 0.001.

The tandem RFP-GFP-LC3 construct was further used to determine whether the effect of 163N was due to autophagic flux suppression. The normal autophagy process causes the reduction of green fluorescent protein (GFP) fluorescence in an acidic lysosome environment, whereas red fluorescent protein (RFP) is more stable under acidic conditions [27]. Therefore, rapamycin (Rap) treatment led to an increase in total puncta, as well as the red-only fluorescence puncta in HEK293 cells (Figure 4A). However, Baf treatment led to an increase in both GFP- and RFP-positive fluorescence puncta (puncta formation with yellow overlay), which was mimicked by 163N treatment (Figure 4A). These yellow overlays represented both autophagosomes and autolysosomes, due to impaired degradation steps, suggesting that 163N suppressed autophagy at a late stage. 

During the late stage of autophagy, autophagosomes fuse with lysosomes to form autolysosomes, where the degradation of the contents occurs. To address whether 163N affected autophagosome-lysosome fusion, we examined the localization of endogenous LC3 with the lysosome membrane marker LAMP1. The compound 163N induced a remarkable increase of LC3 puncta, which were well co-localized with LAMP1 (Figure 4B). This indicated that 163N inhibited autophagy without affecting autophagosome-lysosome fusion. This was similar to the cells treated with Baf, which blocked the degradation of autolysosomes resulting from elevated lysosomal pH (Figure 4B). However, reduced co-localization of LC3 puncta and LAMP1 was detected in Rap-treated cells (Figure 4B). Electron microscopy was further used to show that more autolysosomes (monolayer structures with cellular components), but not autophagosomes, were accumulated under 163N treatment (Figure 4C). In addition, we transfected GFP-LC3[G120] and GFP-LC3 plasmids into ATG4BKO HeLa cells, respectively. As shown in Figure 4D, GFP-LC3 puncta were hard to find in GFP-LC3 transfected ATG4BKO cells, with or without Rap, CQ, or 163N treatments, due to the defect conversion of pro-LC3 to LC3-I. In contrast, cells transfected with GFP-LC3[G120], which can mimic LC3-I, had more dots. However, the level of GFP-LC3 dots in 163N treated cells was still lower than Rap or CQ treated cells, possibly because the lack of autophagy induction (Rap) or autophagosome lysosome fusion inhibition effect (CQ), suggesting that 163N may mainly affect the degradation of the autolysosome. These findings indicated that 163N inhibits autophagy without blocking the fusion between autophagosomes and lysosomes, but increases the accumulation of autolysosomes.

### 2.5. 163N Causes Lysosome Dysfunction

**Figure 5:**
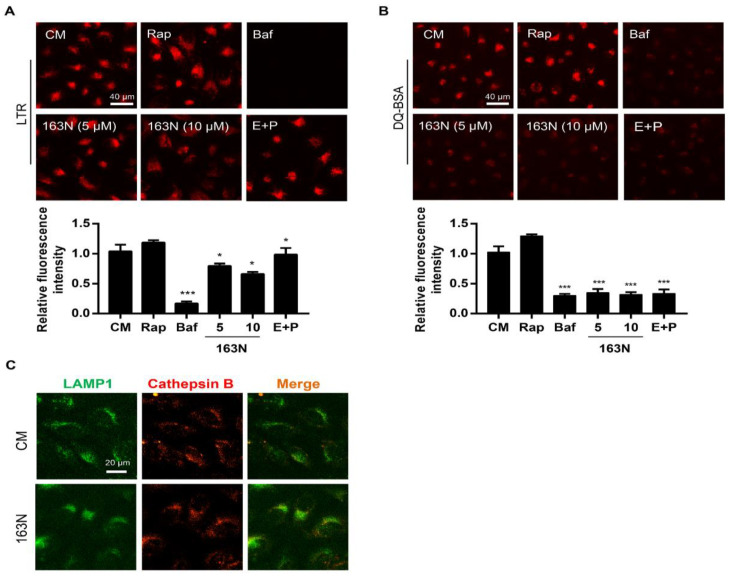
163N causes lysosome dysfunction. (**A**) HeLa cells were treated with Rap (1 µM), Baf (0.5 µM), E64D (25 µM) plus pepstatin A (50 µM) or different concentrations of 163N (5 or 10 µM) for 6 h, then LysoTracker Red (LTR, 25 ng/mL) was used to stain for 30 min, then the relative LTR fluorescent intensity was quantified. (**B**) HeLa cells were firstly incubated with 10 µg/mL DQ-BSA in EBSS medium for 1.5 h and then treated with Rap (1 µM), Baf (0.5 µM), E64D (25 µM) plus pepstatin A (50 µM) or different concentrations of 163N (5 or 10 µM) for another 6 h, then the relative DQ-BSA fluorescent intensity was quantified. (**C**) HeLa cells were treated with or without 163N (10 µM) for 6 h, followed by immunostaining using cathepsin B and LAMP1 antibody. The colocalization of cathepsin B and LAMP1 was measured. Data are presented as mean ± SEM from three individual experiments. * *p* < 0.05, *** *p* < 0.001.

There was an increased accumulation of LC3 on autolysosomes caused by 163N and this could be associated to elevated pH or impaired protease activity in lysosomes, or defective degradation of LC3-PE on autophagosomal structures, due to the suppression of ATG4B activity [25]. As shown in Figure 5A, treating cells with 163N caused a significant reduction in acidic vesicles, and this was similar to the Baf-treated cells, although weaker than the effect of Baf. However, the red fluorescence signal of LysoTracker Red (LTR) in Rap or E64D plus pepstatin A-treated cells was stable. Therefore, 163N affected the acidity of the lysosome but was not as strong as Baf. Next, we monitored the degradation of lysosome during 163N treatment using DQ-BSA [28]. Under normal lysosomal conditions, proteolysis of DQ-BSA resulted in dequenching and release of red fluorescence in the lysosome. As shown in Figure 5B, compared with the complete medium or Rap-treated cells, both Baf, E64D plus pepstatin A, and 163N treatment resulted in nearly no dequenching of DQ-BSA, indicating that 163N could significantly inhibit the proteolytic activity of the lysosome. Next, we want to know whether treatment with this compound could cause the release of the lysosomal content, so we analyzed the colocalization of cathepsin B and LAMP1. As shown in Figure 5C, the co-localization of cathepsin B and LAMP1 was observed in 163N treated cells, similar to the complete medium group cells, suggesting that 163N did not produce lysosomal membrane permeabilization. Together, these observations indicated that 163N treatment could cause lysosome dysfunction. 

### 2.6. 163N Suppresses the Growth of Cancer Cells in Vitro

**Figure 6:**
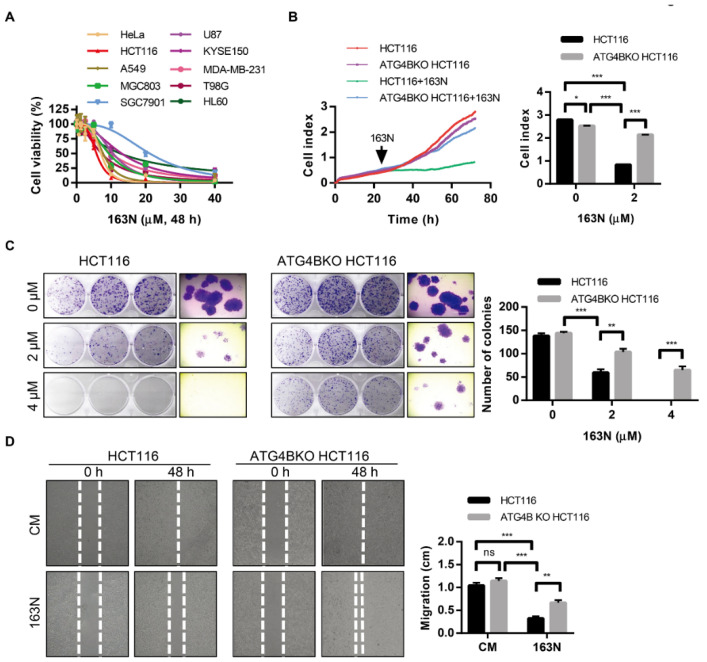
163N suppresses the growth of colorectal cancer cells in vitro. (**A**) HeLa, HCT116, A549, MGC803, SGC7901, U87, KYSE150, MDA-MB-231, T98G, and HL60 cells were treated with various concentrations of 163N, and CCK-8 assay was performed to assess cell proliferation. (**B**) HCT116 and ATG4BKO HCT116 cells (2500 per well) were seeded before treated with 163N (2 µM), and 0.1% Dimethyl-sulfoxide (DMSO) was used as a control. The real-time cellular analysis (RTCA) plots were generated using RTCA software 1.1.2, and the cell index was analyzed with GraphPad 7.0 software. (**C**) HCT116 and ATG4BKO HCT116 cells were treated with a series of concentrations of 163N. Colony formation assay was then monitored by crystal violet staining. The number of the clones were counted and quantified. (**D**) HCT116 and ATG4BKO HCT116 cells were treated with or without 163N (10 µM) for 48 h, wound healing was recorded (left) and quantitatively analyzed (right). Data are presented as mean ± SEM from three individual experiments. * *p* < 0.05, ** *p* < 0.01, *** *p* < 0.001.

Previous studies have shown that autophagy supplies ATP to maintain cellular biosynthesis in tumors; there are also studies showing that autophagy inhibition through genetic or pharmacological interventions is an effective way of overcoming treatment resistance [8,29,30]. To investigate whether 163N-mediated autophagy inhibition could affect cancer cell survival, cell viability was estimated by CCK-8 assay. As shown in Figure 6A, 163N significantly reduced the cell viability in different cancer cells, including HeLa (IC50 = 7.06 µM), HCT116 (IC50 = 5.76 µM), A549 (IC50 = 7.68 µM), MGC803 (IC50 = 9.33 µM), SGC7901 (IC50 = 21.16 µM), U87 (IC50 = 10.59 µM), KYSE150 (IC50 = 13.03 µM), MDA-MB-231 (IC50 = 9.99 µM), T98G (IC50 = 7.23 µM), as well as HL60 (IC50 = 12.63 µM). In addition, we found that 163N could reduce the cell viability of normal cells (L02 and MRC-5 cells) at a higher concentration (40 µM), but tumor cells were more sensitive to 163N than normal cells at a lower concentration (10 µM) (Figure S3). As expected, 163N exhibited about five times higher cytotoxicity than XOS00163, XOS00201, and XOS00207 for HeLa and HCT116 cells (Figure 6A and Table 1). To further examine the effect of 163N on cell proliferation, HCT116 cells were used to perform real-time cellular analysis (RTCA) assays. RTCA plots were generated using RTCA software 1.1.2. As shown in Figure 6B, 2 µM of 163N had significant cytotoxic effects on colorectal cancer HCT116 cells. We then generated ATG4B-deficient HCT116 cells by CRISPR/Cas9 system (Figure S4A,B). Supported by previous findings that ATG4B was required for cell cycle regulation [16,31]. Knockout of ATG4B in HCT116 cells resulted in lower cell proliferation rate than the wild type HCT116 cells (Figure 6B). The study also found that ATG4BKO HCT116 cells were less sensitive to 163N treatment, compared with wild type HCT116 cells (Figure 6B), and similar results were also observed using colony formation assays (Figure 6C). In addition, inhibition of autophagy either by CQ (IC50 = 14.2 µM) or by genetic depletion of ATG16L1 (IC50 = 12.37 µM) could also reduce the cytotoxicity induced by 163N (Figure S5). These results imply that 163N might target autophagy to reduce tumor viability. Moreover, we could also find that inhibition of autophagy by 163N exhibited a much stronger inhibition effect on tumor cell viability than the genetic deletion of ATG4B (Figure 6B,C), implying that only ATG4B inhibition is likely not sufficient to suppress tumor growth. An evaluation of the potential roles of 163N on in vitro motility of wild type HCT116 cells and ATG4BKO HCT116 cells was performed as well. The findings showed that there was no significant difference that was observed in the migration of wild type HCT116 cells and ATG4BKO HCT116 cells under normal medium (Figure 6D). However, 163N treatment significantly inhibited the migration of wild type HCT116 cells, whereas the minimal effect was observed in ATG4BKO HCT116 cells (Figure 6D). Moreover, inhibition of autophagy by 163N had a stronger inhibition effect on tumor cell migration than just genetic deletion of ATG4B, indicating that single ATG4B knockout is not enough to inhibit tumor cell migration. Together, 163N might exert its antitumor effect through targeting autophagy, and only deletion of ATG4B is not sufficient to suppress tumor growth, which may explain why the dual functional autophagy inhibitor 163N can potently inhibited the growth of colorectal cancer cells. 

### 2.7. 163N Suppresses the Growth of Colorectal Cancer Cells in Vivo

**Figure 7:**
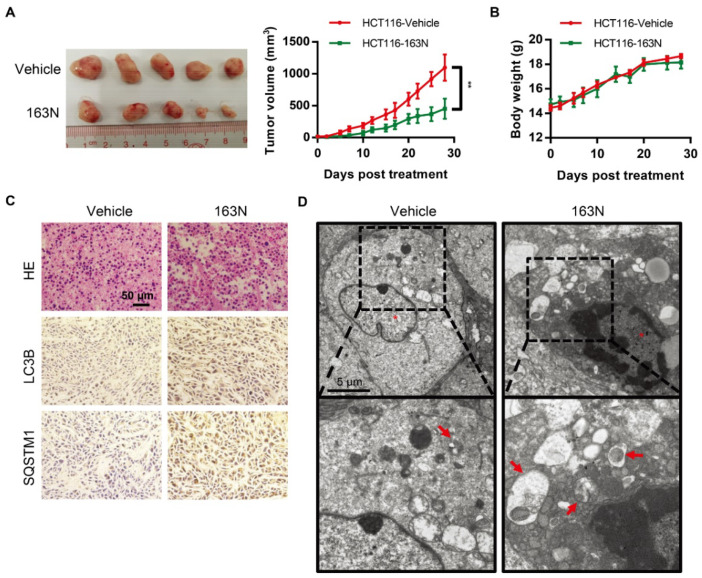
163N inhibits tumor growth in a HCT116 mouse xenograft model. (**A**) HCT116 cells (3 × 106) were injected subcutaneously into the right flank of the immunodeficient nu/nu female mice. Seven days after tumor cell implantation, mice with palpable tumors were divided randomly into two groups and injected intraperitoneal on Monday, Wednesday, and Friday with either corn oil vehicle or 163N (50 mg/kg). Tumor volumes were calculated by the length and width measured by Vernier calipers every 2 d. (**B**) Body weight changes of mice during 28 d of exposure. (**C**) Tumor tissues were sectioned and subjected to H&E staining and immunohistochemistry for evaluating histological morphology, and expression of LC3B and SQSTM1. Scale bar: 50 µm. (**D**) TEM was used to detect the ultrastructure of the representative tumor tissues. Red arrows represent normal autolysosome structures. Data are presented as mean ± SEM. ** *p* < 0.01 vs VC.

Next, the effect of 163N in suppressing the growth of colorectal cancer cells using a mouse xenograft model was investigated. This was achieved by introducing HCT116 cells into immunodeficient mice. After 7 days, when the tumors were palpable, the mice were randomized assigned to 2 groups, and treated with vehicle corn oil or 163N. As shown in Figure 7A, tumor growth in vehicle-treated mice increased dramatically. However, tumor growth in 163N-treated mice was less prominent, and there were no significant changes in body weight among the vehicle control and 163N treatment groups (Figure 7B). There were also no significant changes in weight index (Figure S6A) and morphology (Figure S6B) of the heart, liver, spleen, lung, and kidney among vehicle control and 163N treatment groups. To evaluate whether 163N treatment changed the morphology of HCT116 cells in vivo, hematoxylin-eosin (H&E) staining was performed. As shown in Figure 7C (top panels), tumor sections from mice treated with 163N exhibited abnormal cell arrangement and signs of necrosis with infiltration of inflammatory cells. Then, the increased levels of LC3B and SQSTM1 was detected by immunohistochemistry as shown in Figure 7C, suggesting that 163N might inhibit autophagy in vivo. Then, transmission electron microscopy (TEM) was used. As shown in Figure 7D, the TEM studies showed that the number of autophagic vacuoles (especially autolysosomes) was higher in the tumor tissues of the 163N-treated mice compared with the vehicle control group. These findings indicate that 163N-mediated inhibition of tumor growth could be associated with autophagy inhibition. 

## 3. Discussion

There is evidence indicating that autophagy acts to promote tumor survival and growth in established cancers at the later stages from stressful conditions, such as hypoxia, therapeutic stresses, or nutrient deprivation [5,32,33,34]. At present, inhibitors targeting autophagy are mainly lysosomal inhibitors and inhibitors against other specific autophagy targets, such as ULK1 [35], class III PI3K (VPS34) [35], BECN1 and ATG4B have also been reported [17]. Early-stage targets participate in the formation of autophagosomes such as ULK1, VPS34, and BECN1. Whereas late-stage autophagy usually refers to the fusion between autophagosomes and lysosomes, or the degradation of autolysosomes. Research indicates that autophagosomal structures serve as scaffolds to induce apoptosis and necroptosis [36,37]. Therefore, inhibitors targeting late stage of autophagy might be better for cancer treatment. 

As discussed above, with the exception of CQ and its derivatives, none of these reported inhibitors of ATG protein have approved for clinical cancer treatment. One of the main concerns when using these lysosomotropic agents is that they may function independent of autophagy, which may lead to some unfavorable side effects. Therefore, it is of great theoretical and practical significance to develop novel and efficient autophagy inhibitors as anticancer drugs. ATG4 is a key protein of autophagy signal pathway. Of the four ATG4 family proteins, ATG4B is 1500-fold more efficient in the catalytically activity towards LC3B than the other three ATG4s [38]. Studies have shown that deletion of ATG4B or overexpression of a catalytic-negative mutant could arrest autophagy [39,40]. Therefore, targeting ATG4B provides an alternative strategy to inhibit autophagy. The clinical association of biomarkers in colorectal cancer have been reported [41]. A previous study has shown that high ATG4B expression intensity was almost correlated with late tumor stages (II-IV) of colorectal cancer patients [16]. Recent studies have demonstrated that using small molecule inhibitors of ATG4B could suppress the growth of colorectal cancer cells [25], or sensitize colorectal cancer cells to chemotherapy [42]. These results indicate that ATG4B may serve as a novel biomarker in colorectal cancer. 

In this study, we provide a new therapeutic strategy and aim to identify a dual functional molecule that simultaneously targets ATG4B and lysosome to inhibit autophagy, potentially serving as promising anti-tumor agents. Currently, a series of ATG4B inhibitors have been reported. For example, NSC185058 was reported as an ATG4B inhibitor, which could suppress the growth of osteosarcoma tumors and enhance the anti-cancer activity of radiation therapy [17,43]. Tioconazole was also identified to be a potent ATG4B inhibitor, which could diminish autophagic flux and enhance the anti-tumor effect of chemotherapeutic drugs [42]. S130 has been shown to inhibit autophagy flux and suppress the growth of colorectal tumors [25]. FMK-9a was shown to be the most potent ATG4B inhibitors so far, but it could neither inhibit autophagy nor reduce tumor cell viability [44,45]. These findings demonstrate that the anti-canner effect of these ATG4B inhibitors may not be correlated with the inhibition efficiency of ATG4B catalytic activity. In the current study, we employed a FRET-based assay to screen an in-house new drug library of 560 compounds. Based on the screening, chemicals with a 2-phenylbenzofuran structure exhibited a strong inhibition effect on ATG4B and cancer cell viability (Table 1), suggesting the structure with a 2-phenyl benzofuran might be a good pharmacophore to develop ATG4B inhibitors for cancer therapy. Our previous results implied that compounds with a 6-methylpyrimidine-2,4-diamine structure could cause lysosome inhibition [24]. Thus, we synthesized a series of compounds with a 6-methylpyrimidine-2,4-diamine structure (Figure S1A), and confirmed that these chemicals are strong lysosomotropic agents (Figure S1B,C). Thus, 163N bearing the structure of both 2-phenylbenzofuran and 6-methylphenylpyrimidine-2,4-diamine, might be a potential dual functional autophagy inhibitor for the potential use of cancer treatment. 

Taking account of the complexity of the pathogenesis of cancer, single-target drugs are prone to drug resistance. Considering drug combination usually leads to many shortcomings, such as complicated pharmacokinetic properties, complex dose settings, and drug-drug interactions. Therefore, it is urgent to develop more effective therapeutic strategies to overcome these drawbacks, and multi-functional molecules can effectively solve the above disadvantages. Our results suggest that compared to XOS00163, XOS00201, and XOS00207, 163N shows a slightly lower activity on ATG4B, but has a stronger anti-cancer effect. And compared to Baf, 163N possessed a mild lysosomotropic effect (Figure 5A), whereas the lysosomotropic effect of Baf was weaker than L1, L2, L3, L4, L5, and L6 (Figure S1B). These findings suggest that 163N not only retains the inhibition effect on ATG4B and lysosome, but also improves the anti-cancer effect, and reduces lysosome alkalization in cells. 

For further clarification the possible mechanism of 163N on autophagy, we examined the action of 163N on LC3-II formation, autophagy flux, autophagosome-lysosome fusion, and the degradation of autolysosome. Results show that 163N inhibited autophagic flux and caused the accumulation of autolysosomes without affecting autophagosome-lysosome fusion. It has been reported previously that the accumulation of LC3-II on the autolysosome may be associated to elevated pH or impaired protease activity in lysosome, or defective degradation of LC3-PE on autophagosomal structures, due to the suppression of ATG4B activity [25]. Results from LTR staining and DQ-BSA staining show that 163N-mediated blockage of degradation of autolysosome might be due to lysosomal pH elevation and impaired proteolytic activity (Figure 5A,B), but does not affect lysosomal membrane permeabilization (Figure 5C). However, the exact target of 163N acting on lysosome to cause lysosome dysfunction remains to be explored. Further studies in Figure 6B show that knockout of ATG4B could reduce the sensitivity of tumor cells to 163N treatment, implying that 163N might partially target ATG4B in cells, and this factor may also contribute to the increase of autolysosome induced by 163N treatment. In addition, blocking autophagy by pharmacological (CQ) or genetic approaches (knockout of ATG16L1) also partially attenuated the cytotoxicity induced by 163N (Figure S5), suggesting that autophagy is required for 163N induced cytotoxicity. However, whether this cell protection effect is because the loss of ATG4B, or autophagy deficiency, or both, still needs to be further investigated. These results indicate that 163N functions as an autophagy inhibitor to exert its antitumor effect. 

As described above, autophagy inhibition could be therapeutically effective for cancer control. Our results suggest that the anti-cancer effect of 163N is consistent with its ability to suppress autophagy. 163N suppressed tumor growth in vitro and in vivo. Surprisingly, no obvious changes in animal weight was observed in 163N-treated mice (Figure 7B). Also, the lack of any obvious changes in weight and morphologies of heart, liver, spleen, lung and kidney were observed in mice treated with 163N (Figure S4A,B). These results indicate that 163N might have a good safety performance. Together, our findings suggest that the inhibition of autophagy by 163N might be an efficient way to treat colorectal cancer.

## 4. Materials and Methods

### 4.1. Synthesis of 163N

The graphical synthetic route of compound 163N is described in Figure 1. The solution of K2CO3 (30 mM) and 4-Nitrobenzyl-bromide (10 mM) in DMF was added 2-Hydroxybenzaldehyde (10 mM). The reaction mixture was stirred at 150 °C for 12 h under an inert atmosphere. After cooling to room temperature, ethyl acetate and water were used to extract the mixture. Then, brine was used to wash the combined organic layer, followed by anhydrous Na2SO4 drying and reduced pressure concentrating. Finally, flash silica gel column chromatography (1:10 ethyl acetate/petroleum ether) was used to purify the crude reaction mixture to get the 2-(4-nitrophenyl) benzofuran (75% yield).

The solution of 2-(4-nitrophenyl) benzofuran (8.5 mM) in methanol was added Raney Nickel (M/M = 10%, 250 mg) followed by hydrazine hydrate slowly at 0 °C. The reaction mixture was stirred 3 h at room temperature. After completion of reaction, the mixture was filtered with kieselguhr and washed with methanol, and the filtrate was concentrated under reduced pressure. The crude product (100% yield) was directly in the next step.

The solution of 2, 4-dichloro-6-methylpyrimidine (20 mM) and Et3N (24 mM) in ethanol at 0°, 4-methoxyaniline (20 mM) was added. The reaction mixture was stirred overnight at room temperature. After completion of reaction, the mixture was filtered and the filtrate was concentrated under reduced pressure. The crude product was purified by flash silica gel column chromatography (1:10 ethyl acetate/petroleum ether) to afford the compound 2-chloro-N-(4-methoxyphenyl)-6-methylpyrimidin-4-amine (75% yield).

In a seal tube, the solution of 4-(benzofuran-2-yl) aniline (3 mM), 2-chloro-N-(4-methoxyphenyl)-6-methylpyrimidin-4-amine (3 mM) in t-butanol (30 mL) was added, concentrated hydrochloric acid (catalytic amount). The reaction mixture was stirred at 150 °C for 16 h under an inert atmosphere. After cooling to room temperature, the mixture was filtered and washed with t-butanol. The filter cake was diluted with dichloromethane and dilute ammonia water. The organic layer was washed with water and brine, dried with Na2SO4 and concentrated under reduced pressure. The crude product was purified by flash silica gel column chromatography (1:100 methanol/ dichloromethane) to afford the compound 163-N (83% yield), which was characterized by 1H- and 13C-NMR, and LC-MS.

### 4.2. Antibodies and Reagents

Antibodies used in this study were as follow: ATG4B (15131-1-AP) was from Proteintech (Wuhan, China), ATG5 (8540) and FIP200 (12436) were from Cell Signaling Technology (Danvers, MA, USA), GAPDH (sc-365062) was from Santa Cruz Biotechnology (Dallas, TX, USA), LC3B (L7543) and TUBA (T6074) were from Sigma (St. Louis, MO, USA), LAMP1 (H4A3) was from Developmental Studies Hybridoma Bank (Iowa City, IA, USA), SQSTM1/p62 (PM045) was from MBL International (Woburn, MA, USA). Secondary antibodies (35503, 35511, 35553, and 35561) were purchased from Thermo Fisher Scientific (Rockford, IL, USA).

Bafilomycin A1 (Baf, B-1080) was purchased from LC Laboratories (Woburn, MA, USA), as well as rapamycin (Rap, ASW-125). Chloroquine (CQ, A506569) was purchased from Sangon Biotech (Shanghai, China). Z-VAD-FMK (S7023) and staurosporine (SP, S1421) was from Selleckchem (Houston, TX, USA). Dequenched-BSA (DQ-BSA) Red (D12051), LysoTracker Red (LTR, L12492) and Lipofectamine 2000 (11668-019) were purchased from Life Technologies (Bothell, WA, USA). Substrate Ac-DEVD-AFC (CASP-048) for caspase3 activity was from Chinese Peptide Company (Hangzhou, China). Purified ATG4B, substrate ECFP-GATE-16-YFP (FRET-GATE16) for ATG4B were described before [45]. 

### 4.3. Cell Culture and Plasmid Transfection

HeLa, HCT116, GFP-LC3 A549, and ATG4B-deficient HeLa (ATG4BKO-HeLa) cells have been described previously [25,44]. All cells were routinely cultured in Dulbecco’s modified eagle medium (DMEM) medium (Thermo Scientific, Waltham, MA, USA, 11965092) with 10% fetal bovine serum (Gibco, 10270) and standard supplements. All cells were maintained in a 37°, 5% (v/v) CO_2_ incubator. Cells were grown in 12/24 well plates before transfection using Lipofectamine 2000.

### 4.4. Generation of ATG4B Knockout HCT116 Cell Line

The design of gRNAs for human ATG4B has been described previously [25]. Then, HEK293T cells were transfected with lentiCRISPRv2-gRNA, psPAX2 and pMD2.G at a ratio of 4:3:1 to produce lentiviral particles. After 72 h, viral supernatants were collected. HCT116 cells grown in 24-well plate format were transduced with lentivirus for 24–48 h. After that puromycin (1 µg/mL) were added for 2–3 days and then genomic DNA were extracted. Design PCR primers upstream and downstream of the knockout site: -5′ TGCTGTAAATGGCCTCTTTCG, -3′ CAAGTCACAAGTTATGGCAGAG. Perform PCR using genomic DNA as a template, and use PCR products for sequencing. Single-cell clones were then isolated by multiple dilution in 96-well plates. Single ATG4B deficient clones were detected by western blot.

### 4.5. Immunoblotting Assays

20–30 µg of whole cell lysates were separated by SDS-PAGE to avoid saturation and transferred to PVDF membranes (Millipore, ISEQ00010, Burlington, MA, USA). After blocking, the membrane was incubated with primary antibodies and secondary antibodies. Specific proteins were detected using enhanced chemiluminescence developing agent (Millipore, WBULS0500). Images were taken using Kodak Image Station 4000 (Carestream Health, Rochester, NY, USA).

### 4.6. Immunostaining Assay

Immunostaining assay was performed as described previously [25]. Cells were fixed in 4% paraformaldehyde for 15–20 min followed by permeabilization with 0.1% Triton X-100 for another 15 min. Then, PBS containing 5% bovine serum albumin was used for blocking. Primary antibodies (1:150) was used at 4° for one night and then secondary antibodies (1:500) were applied at room temperature for 1 h. EVOS FL Auto (Life Technologies) was used to get the fluorescence images. At least three optical fields with at least 100 cells were analyzed for quantification.

### 4.7. Transmission Electron Microscopy

Electron microscopy was carried out as described [25]. Briefly, 2.5% glutaraldehyde in 0.1 M phosphate buffer (pH 7.4) was used for fixed cell sample. After 2 h, cells were dehydrated in a graded ethanol series and then embedded. Ultrathin sections of these cells were mounted on copper grids, and then stained. Finally, electron microscope Tecnai G2 20 Twin (FEI, Fremont, CA, USA) was used for visualizing. 

### 4.8. Lysosomal Function Analysis

LTR assay: cells were incubated with LTR (25 nM) in HBSS medium at 37° for 30 min, and then fluorescence microscopy was used for detecting fluorescence intensity.

DQ-BSA Red assay: cells were incubated with 10 µg/mL DQ-BSA Red in EBSS medium at 37° for 1.5 h, and then new medium with or without the indicated compounds were added for another 4 or 6 h, and then fluorescence microscopy was used for detecting the intensity of red fluorescent. 

### 4.9. FRET-Based Assay

A FRET-based assay for detecting ATG4B activity was carried out as described [45]. ATG4A (20 µg/mL) or ATG4B (0.75 µg/mL) was incubated with different concentrations of 163N in 384-well plates at 37° for 30 min. And then FRET-GABARAPL2 (50 µg/mL) was added to the mixture to a total volume of 50 µL. After 30 min, the relative fluorescence units (RFUs) ratio of 527 nm/477 nm was tested. Finally, Graphpad 7.0 (GraphPad Software, La Jolla, CA, USA) was used to calculate the IC50 value of 163N.

### 4.10. Pocket Identification and Molecular Docking

The molecular docking of ATG4B was performed as described [25]. Briefly, the crystal structure of ATG4B was downloaded from the Protein Data Bank (PDB) (www.rcsb.org). Site finder model of MOE.2014 was applied for identifying small molecule-binding pockets. The energy-minimized 3D structure of 163N was obtained by MOE.2014 conformations model. Then, 163N (minimized 3D structure) was docked into the pocket site of 2CY7. The docking results were ranked and the best binding pattern of each compounds were detected. PyMOL software (DeLano Scientific, Palo Alto, CA, USA) were used to obtain the structures of the docking models.

### 4.11. Cell Viability Assay

Cells were seeded and incubated overnight in 96-well plates at a density of 5 × 103 cells/well. Then, adherent cells were treated with a series of diluted concentrations of 163N for 48 h. A total of 10 µL CCK-8 were added to each well for additional 1 h and cell viability was measured by CCK-8 assay. Absorbance was measured at 450 nm. The IC50 values were calculated by GraphPad Prism 7 (GraphPad Software Inc., San Diego, CA, USA). 

### 4.12. Real-Time Cellular Analysis (RTCA)

An E-Plate 16 (ACEA Biosciences, Hangzhou, China) was prepared with 50 µL of DMEM medium added to each well to do a background measurement in the xCELLigence instrument (ACEA Biosciences, China). 100 µL of passaged cell suspensions containing 2.5 × 10^3^ cells were added to each well and then cells were settled on the base of the well for 30 min at room temperature. The plate was inserted into the RTCA system (housed within the incubator) and cell index (CI) values were assessed every 5 min over the following 72 h. After 24 h of cell seeding, the assay was paused and 2 µM 163N was added to each well. Then, the plate was placed in the station again and the analysis was restarted. After completing a total of 72 h of analysis, the data were analyzed with GraphPad 7.0 software.

### 4.13. Colony Formation Assay and Wound Healing Assay

Cells were seeded at a density of 500 cells/well in 6-well plates and incubated overnight. Then, adherent cells were treated with a series of diluted concentrations of 163N. The culture medium was changed every 3 d. After about 2 weeks, 4% paraformaldehyde was used to fix the clones and then 0.2% crystal violet (in 10% formalin) was used for dyeing. The stained cells were washed by PBS and colonies over 1 mm were counted from 3 independent experiments.

Cells were seeded in 12-well plates. After the cells grew to confluence, wounds were made by sterile pipette tips. The cells were photographed at the beginning of wound formation. Cells were washed with PBS and refreshed with medium with or without 10 µM of 163N and photographed after 48 h of incubation at the same field of view. The distance of cell migration was calculated from three independent experiments.

### 4.14. Tumor Xenograft Studies

The animal experimental procedures were approved by the Research Ethics Committee of Sun Yat-sen University (Guangzhou, China), ethic code: SYSU-IACUC-2018-000134. Four-week-old immunodeficient mice (nu/nu, female) were raised in a pathogen-free environment in the Experimental Animal Center of Sun Yat-sen University. Human colorectal cancer HCT116 cells (3 × 10^6^) were injected subcutaneously into the right flank of the animals. Seven days after tumor cell implantation, mice with palpable tumors were divided randomly into two groups and injected intraperitoneal on Monday, Wednesday, and Friday of the following treatments: (1) vehicle control (corn oil); (2) 163N (50 mg/kg body weight) dissolved in corn oil. Tumors were measured using calipers and the tumor volume (mm^3^) was calculated as follows: V = (L × W2)/2, L and W represents tumor length and width, respectively. At the end of the experiment (5 weeks after tumor implantation), the mice were given 200 µL of pentobarbital sodium (1%) by i.p. 30 min before sacrifice, tumors and organs were collected and weighed. 

### 4.15. Statistical Analysis

Data were reported as mean ± SEM from at least three independent experiments unless otherwise specified. Statistical analyses were determined using the Student 2-tailed *t* test. Values of * *p* < 0.05 were considered as being significant.

## 5. Conclusions

In summary, we have generated a novel dual-effective autophagy inhibitor 163N that targets both ATG4B and lysosome to effectively inhibit tumor growth in vitro and in vivo. In addition, the anti-cancer effect of 163N was consistent with its ability to suppress autophagy. Therefore, 163N might be a promising lead compound for further druggability evaluation and anti-tumor application. Furthermore, these studies open new venues for developing multi-target drugs to improve potential cancer therapeutics and new strategy for future autophagy-based multi-functional drug discovery.

## Figures and Tables

**Table 1:** List of the screened ATG4B inhibitors.

Compound ID	Structure	ATG4B-IC_50_ (μM)	HeLa-IC_50_ (μM)	HCT116-IC_50_ (μM)	HL60-IC_50_ (μM)
XGS00025	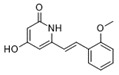	29.19	>100	>100	>100
XGS00031	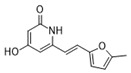	13.03	>100	>100	>100
XGS00043	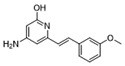	36.31	>100	>100	>100
XGS00046	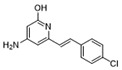	39.14	>100	>100	>100
XGS00048	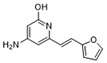	13.81	>100	>100	>100
XGS00119	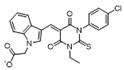	7.37	>100	>100	>100
XOS00117	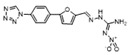	6.65	>100	>100	>100
XOS00125	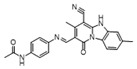	40.64	>100	>100	>100
XOS00149	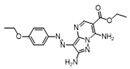	44.03	>100	>100	>100
XOS00154	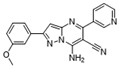	40.71	>100	>100	>100
XOS00163	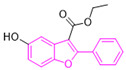	6.2	31.98	22.97	31.7
XOS00176	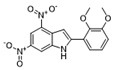	23.81	>100	>100	>100
XOS00201	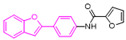	8.98	25.64	19.62	>100
XOS00207	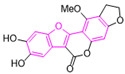	2.31	87.14	17.63	60.7
163N	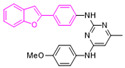	17.82	7.06	5.76	12.63

Purple represents a 2-phenylbenzofuran structure.

## Supplementary Materials

The following are available online at https://www.mdpi.com/2072-6694/12/6/1523/s1, Figure S1: Compounds with a 6-methylpyrimidine-2,4-diamine structure could cause lysosome inhibition, Figure S2: 163N could inhibit autophagy flux, Figure S3: Tumor cells HeLa, HCT116, A549, MGC803, U87, KYSE150, MDA-MB-231, T98G, HL60, and normal cells HL60 and MRC-5 cells were treated with 0–40 µM of 163N for 48 h, and CCK-8 assay was performed to assess cell viability, Figure S4: The identification of ATG4B knockout HCT116 cells, Figure S5: 163N induces cell death through targeting autophagy, Figure S6: 163N could inhibit tumor growth in vivo without obvious toxicity to organs, Figure S7: The uncropped Western blots and molecular weight markers of the figure 3 in the main text.
