# Supplementary material for: New Anti-Cancer Strategy to Suppress Colorectal Cancer Growth Through Inhibition of ATG4B and Lysosome Function
Source: Cancers (Basel). 2020 Jun 10;12(6):1523. doi: 10.3390/cancers12061523 (PMC7352571; doi:10.3390/cancers12061523)
Supplement: Supplementary file 1 [file cancers-12-01523-s001.pdf]

cancers

Supplementary Materials: New Anti-Cancer Strategy to Suppress Colorectal Cancer Growth Through Inhibition of ATG4B and Lysosome Function

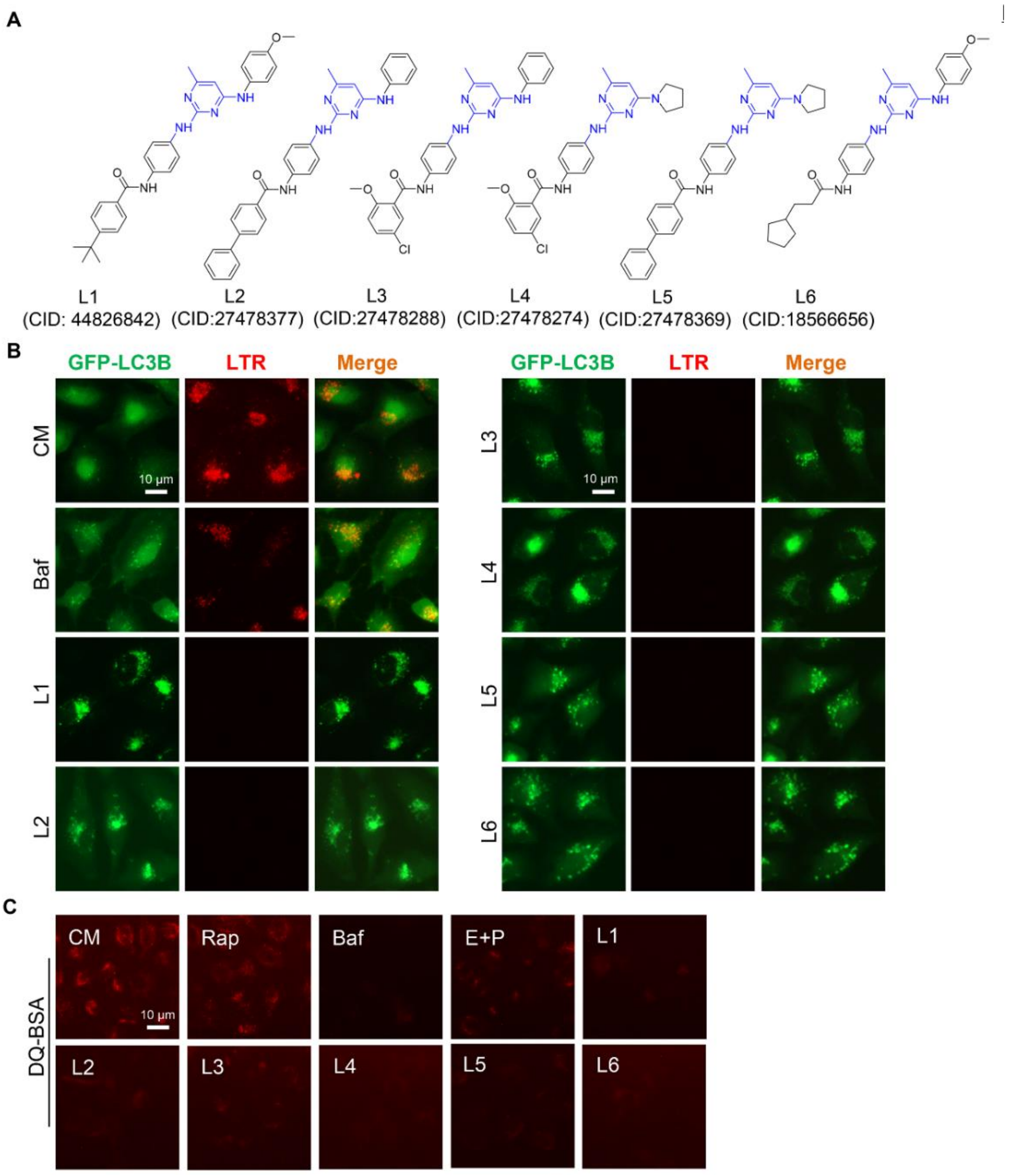

Figure S1. Compounds with a 6-methylpyrimidine-2,4-diamine structure could cause lysosome inhibition. (**A**) The structure of the representative lysosome inhibitors. (**B**) GFP-LC3B HeLa cells were treated with Baf (0.5 μM) or 5 μM of different lysosome inhibitors for 2 h, then LysoTracker Red (LTR, 25 ng/mL) was used and the relative LTR fluorescent intensity was measured. (**C**) HeLa cells were firstly treated with 10 μg/mL DQ-BSA for 1.5 h, and then treated with Rap (1 μM), Baf (0.5 μM), E64D (25 μM) plus pepstatin A (50 μM), or 5 μM of different lysosome inhibitors for 4 h.

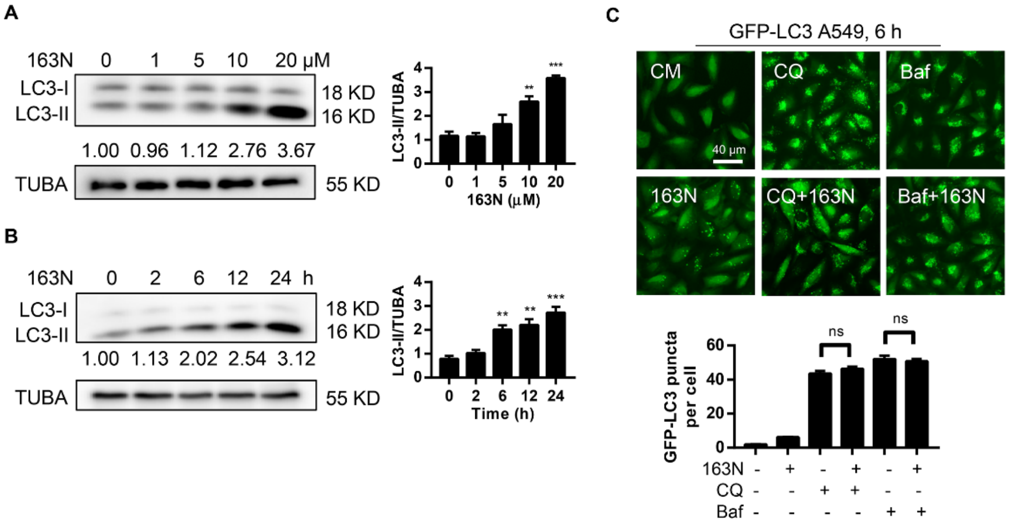

Figure S2. 163N could inhibit autophagy flux. (**A**) HeLa cells treated with different concentrations of 163N (1, 5, 10, 20 μM) for 6 h were analyzed by western blotting for endogenous LC3B. The ratio of LC3B-II/TUBA was calculated using ImageJ software. (**B**) HeLa cells treated with 163N (10 μM) over a certain time course were analyzed by western blotting for endogenous LC3B. The ratio of LC3B-II/TUBA was calculated using ImageJ software. (**C**) A549 cells expressing GFP-LC3B were treated by 163N (10 μM) with or without CQ (40 μM) or Baf (0.5 μM) for 6 h, then the distribution of GFP-LC3B was examined. The number of GFP-LC3B dots was quantified. Data are presented as mean ± SEM from three individual experiments. ** $p < 0.01$, *** $p < 0.001$.

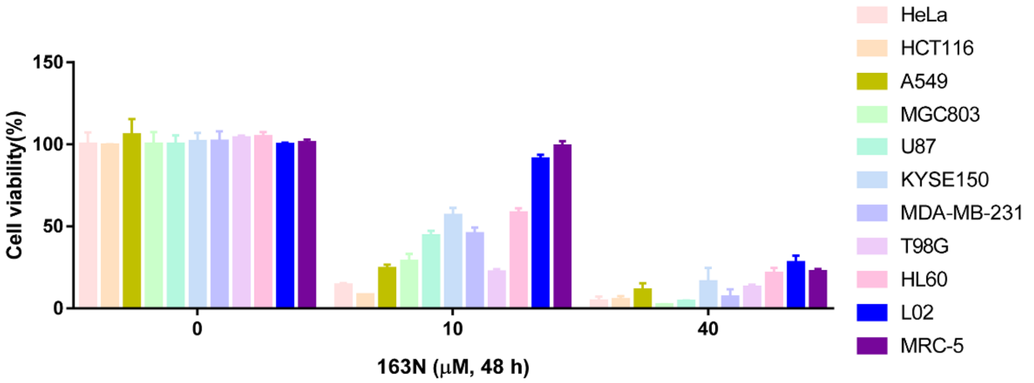

Figure S3. Tumor cells HeLa, HCT116, A549, MGC803, U87, KYSE150, MDA-MB-231, T98G, HL60, and normal cells HL60 and MRC-5 cells were treated with 0-40 μM of 163N for 48 h, and CCK-8 assay was performed to assess cell viability.

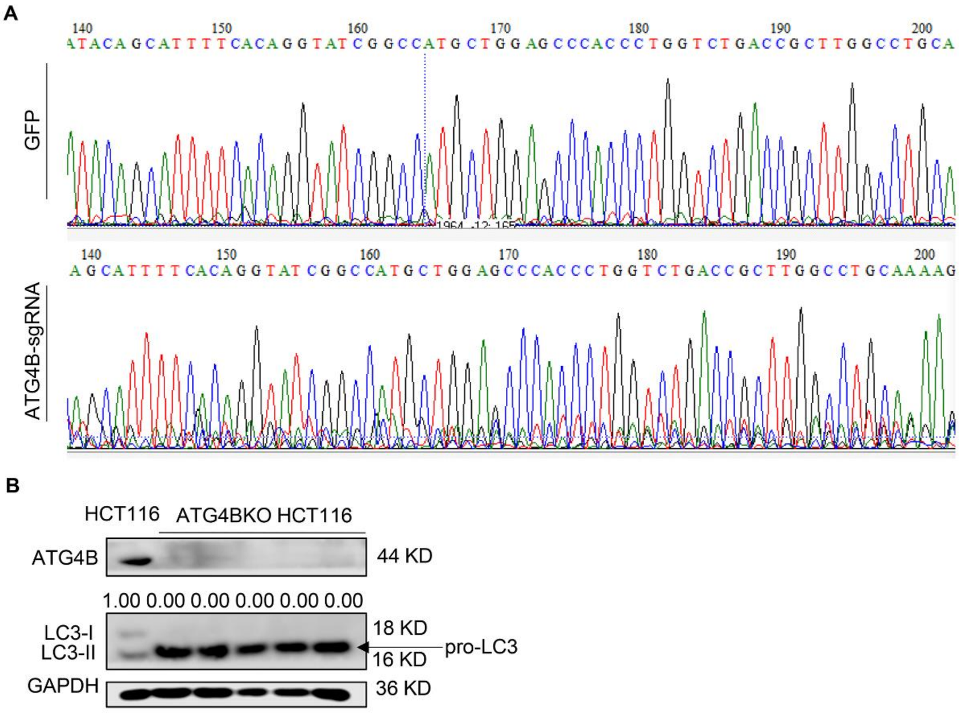

Figure S4. The identification of ATG4B knockout HCT116 cells. (**A**) The genomic DNA of HCT116 cells in the control group transfected with GFP-sgRNA and the experimental group transfected with ATG4B-sgRNA was used as a template for PCR, and the PCR products were used for sequencing. (**B**) Protein levels of ATG4B and LC3B in the control and ATG4BKO cells.

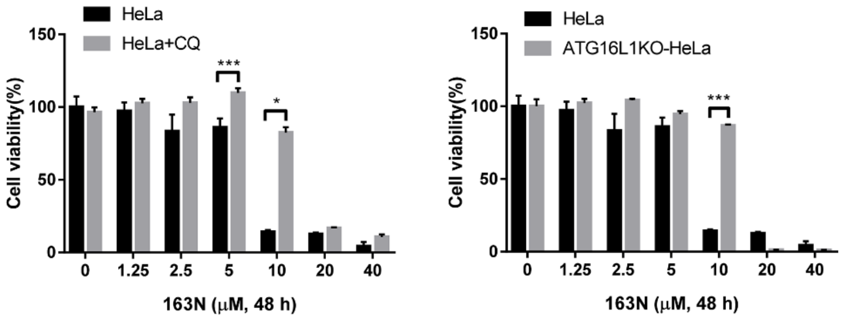

Figure S5. 163N induces cell death through targeting autophagy. (**A**) Hela cells were treated with various concentrations of 163N in the presence or absence of 10 μM CQ, and CCK-8 assay was performed to assess cell proliferation. (**B**) Hela cells or ATG16L1 KO Hela cells were treated with various concentrations of 163N, and CCK-8 assay was performed to assess cell proliferation. Data are presented as mean ± SEM from three individual experiments. * $p < 0.05$, *** $p < 0.001$.

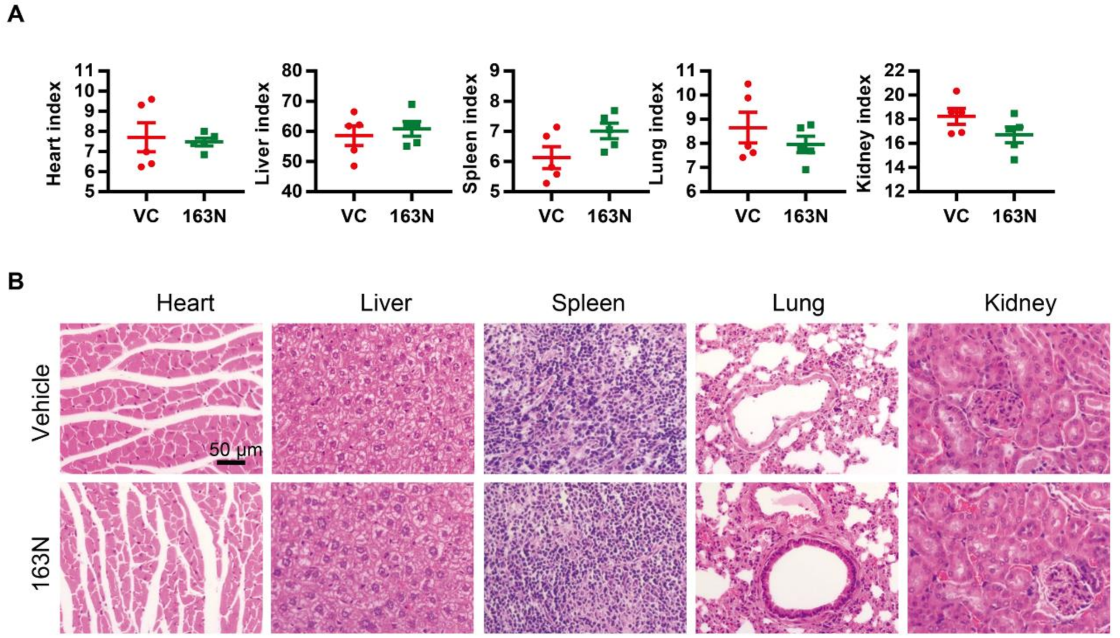

Figure S6. 163N could inhibit tumor growth in vivo without obvious toxicity to organs. (**A**) Heart index, liver index, spleen index, lung index and kidney index were calculated from vehicle and 163N-treated groups. (**B**) The representative mice organs from vehicle and 163N-treated groups were fixed, embedded, sectioned and stained with H&E.

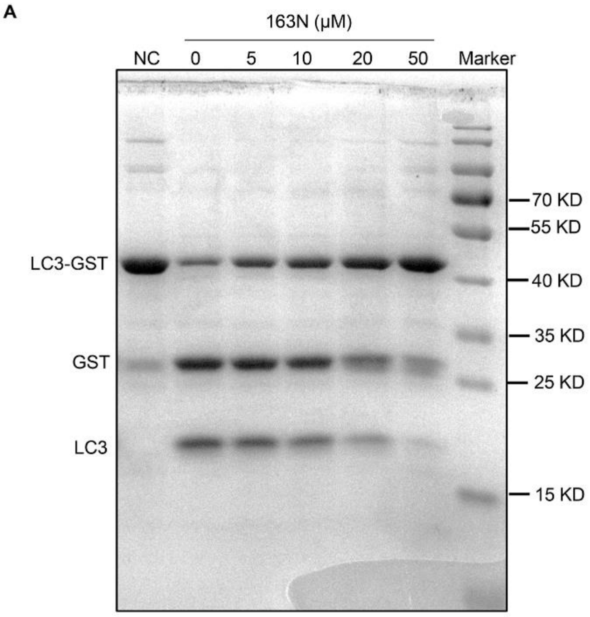

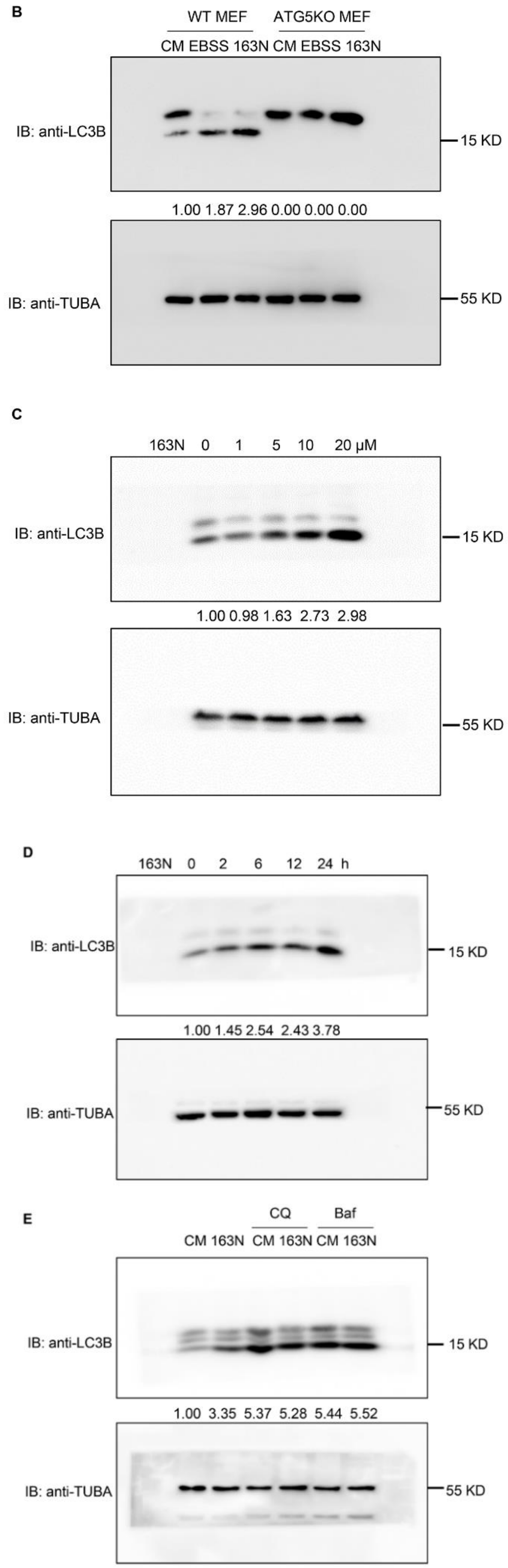
B
WT MEF
ATG5KO MEF
CM EBSS 163N CM EBSS 163N
IB: anti-LC3B
15 KD
1.00 1.87 2.96 0.00 0.00 0.00
IB: anti-TUBA
55 KD
C
163N 0 1 5 10 20 μM
IB: anti-LC3B
15 KD
1.00 0.98 1.63 2.73 2.98
IB: anti-TUBA
55 KD
D
163N 0 2 6 12 24 h
IB: anti-LC3B
15 KD
1.00 1.45 2.54 2.43 3.78
IB: anti-TUBA
55 KD
E
CQ
Baf
CM 163N CM 163N CM 163N
IB: anti-LC3B
15 KD
1.00 3.35 5.37 5.28 5.44 5.52
IB: anti-TUBA
55 KD

F

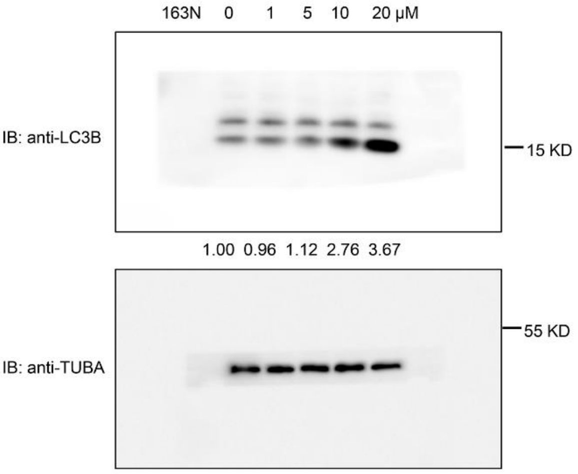

G

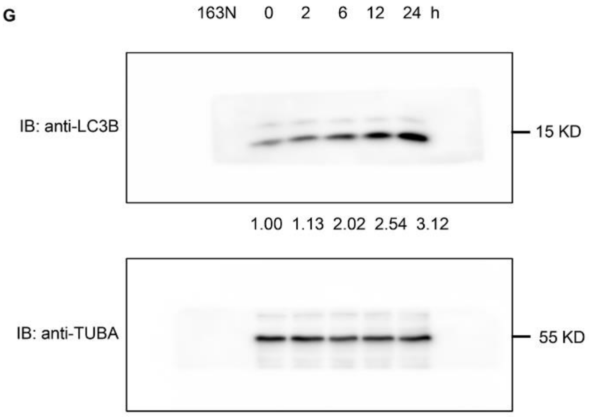

H

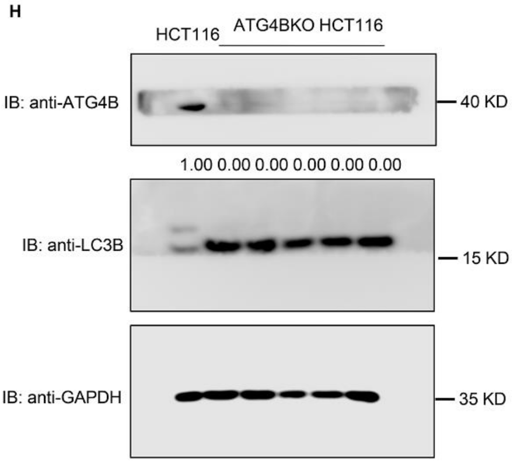

Figure S7. Uncropped Western blots figures. (**A**) The uncropped SDS-PAGE figure of figure 2D. (**B**-**E**) The uncropped Western blots figures of figure 3B-E (**F**-**G**) The uncropped Western blots figures of figure s2A-B. (**H**) The uncropped Western blots figures of figure s4B. The LC3-II or ATG4B intensities in relation to TUBA or GAPDH are shown in these figures.
